# Supplementary material for: The FLARE Score and Circulating Neutrophils in Patients with Cancer and COVID-19 Disease
Source: Cancers (Basel). 2024 Aug 26;16(17):2974. doi: 10.3390/cancers16172974 (PMC11393969; doi:10.3390/cancers16172974)

SUPPLEMENTARY MATERIALS:

The FLARE score and circulating neutrophils in patients with cancer and Covid-19 disease.

Elia Seguí[1,2], Juan Manuel Torres[3], Edouard Auclin[4], David Casadevall[5], Sara Peiro Carmona[3], Juan Aguilar-Company[6], Marta García de Herreros[1,2], Teresa Gorría[2], Juan Carlos Laguna[1,2], Marta Rodríguez[6,7], Azucena González[3], Nicolas Epaillard[4], Javier Gavira[8], Victor Bolaño[3], Jose C. Tapia[8], Marco Tagliamento[9,10], Cristina Teixidó[1,11,22], Hugo Arasanz[12], Sara Pilotto[13], Rafael López-Castro[14], Xabier Mielgo[15], Cristina Urbano[16], Gonzalo Recondo[17], Mar Diaz Pavon[3], Maria Virginia Bluthgen[18], José Nicolas Minatta[19], Lorena Lupinacci[19], Fara Brasó-Maristany[1], Aleix Prat[1,2,20,21,22], Alexandru Vlagea[3] and Laura Mezquita*[1,2,22].

SUPPLEMENTARY METHODS:

Supplementary Table S1: List of participating centers

Hospital Clínic de Barcelona, Barcelona (Spain)
Hospital de Granollers, Granollers (Spain)
Hospital Universitari Vall d'Hebron, Barcelona (Spain)
Hospital del Mar, Barcelona (Spain)
Hospital de la Santa Creu i Sant Pau, Barcelona (Spain)
Hospital Parc Taulí de Sabadell, Sabadell (Spain)
Hospital de Valladolid, Valladolid (Spain)
Hospital Universitario Fundación de Alcorcón, Alcorcón (Spain)
Hôpital Européen Georges-Pompidou, Paris (France)
IRCCS Ospedale Policlinico San Martino, Genova (Italy)
University Hospital of Verona, Verona, (Italy)
Hospital Universitario CEMIC, Buenos Aires (Argentina)
Hospital Alemán, Buenos Aires (Argentina)
Hospital Italiano de Buenos Aires, Buenos (Argentina)

Supplementary Table S2: Detailed Flow Cytometry Panels used for Circulating Immune Cell Phenotyping

Numeration panel: *CD45, CD16, CD3, CD4, CD8, CD19, CD244, CD56;*
Neutrophils panel: *CD15, CD14, CD16, CD10, LOX-1, CD11b, CD64, CD62L*
Monocytes panel*: CD16, CD14, CCR5, CD44, CD71, CD33, HLA-DR, CD163*
T lymphocytes panel: *TCRgd, CD3, CD4, CD8, CD45RA, CCR7, HLA-DR, PD-1.*

Supplementary Table S3: Comprehensive List and Descriptive Profile of Cytokines in the ProcartaPlex Multiplex Assay from Thermo Fisher.

Cytokine	Description
**CD62E (E-selectin)**	Adhesion molecule expressed by endothelial cells upon stimulation with cytokines including TNFalpha and IL-1beta. Induced expression of CD62E during inflammatory conditions is thought to mediate leukocyte rolling including the initial interaction of neutrophils with endothelium. **E-selectin (CD62E) is an endothelial cell specific selectin that is expressed only after activation with proinflammatory cytokines.**
**CD62P (P-Selectin)**	P-selectin protein redistributes to the plasma membrane during platelet activation and degranulation and mediates the interaction of activated endothelial cells or platelets with leukocytes.
**GM-CSF**	Granulocyte-Macrophage colony-stimulating factor stimulates the **development of granulocytes, macrophages,** early megakaryocytes, and eosinophil progenitor cells
**ICAM-1**	ICAM-1 is expressed by activated endothelial cells and detected on epithelial cells, fibroblasts, chondrocytes, B lymphocytes, T lymphocytes (low), monocytes, macrophages, dendritic cells, and neutrophils, with lower levels that increase upon inflammation.
**IFN alpha**	The interaction of IFN-alpha with its receptor component result in the activation of a number of signaling pathways which are mainly involved in i**nnate immune response against viral infection.**
**IFN gamma**	IFN gamma (Interferon gamma, Type II interferon) is a **macrophage** activation factor, and immune interferon that is produced primarily by T-lymphocytes and natural killer cells in response to antigens.
**IL-1 alpha**	IL-1 alpha (Interleukin-1 alpha) is a proinflammatory cytokine expressed by **monocytes, macrophages, and dendritic cells**. IL-1 alpha plays an important role in innate host defense by triggering the production of other proinflammatory cytokines in target cells and initiating acute-phase response.
**IL-1 beta**	Interleukin-1 beta (IL-1 beta) is a proinflammatory cytokine expressed by **monocytes, macrophages, and dendritic cells.**
**IL-4**	Interleukin-4 (IL-4) is a pleiotropic, immune-modulatory cytokine produced by **activated T cells.**
**IL-6**	Interleukin-6 (IL-6) is a multifaceted cytokine produced by various cell types, including fibroblasts, activated T cells, monocytes, macrophages, and endothelial cells. It plays a crucial role in immune responses, such as B-cell maturation into antibody-producing plasma cells. In the context of COVID-19, IL-6 is recognized as a key pro-inflammatory cytokine and a central player in the cytokine storm associated with severe cases of COVID-19
**IL-8 (CXCL8)**	Previous nomenclature for IL-8 includes **neutrophil activating protein** 1 (NAP-1), granulocyte chemotactic protein 1 (GCP-1), monocyte-derived neutrophil-activating peptide (MONAP). The expression and secretion of IL-8 can be induced by diverse inflammatory stimuli in many cells, including macrophages and endothelial cells.
**IL-10**	Interleukin 10 (IL-10, CSIF) is an a**nti-inflammatory cytokine** mainly produced by macrophages (inhibition of macrophage-mediated cytokine synthesis) and Th2 cells. IL-10 functions by inhibiting pro-inflammatory cytokines made by macrophages and regulatory T cells including IFN-gamma, TNF-alpha, IL-2, and IL-3, IL-4, and GM-CSF. IL-10 is also known to suppress antigen presentation on antigen presenting cells, enhances B cell survival, proliferation, and antibody production.
**IL-12p70**	IL-12 is mainly produced by monocytes, macrophages, and dendritic cells in response to bacterial products such as lipopolysaccharides (LPS), to intracellular pathogens or upon interaction with activated T cells. Studies have established that IL-12 also plays a key role in the development of Th1 responses, leading to IFN-g and IL-2 production. These cytokines can in turn promote **T-cell responses and macrophage activation.**
**IL-13**	IL-13 down-regulates macrophage activity, thereby inhibiting the production of pro-inflammatory cytokines and chemokines.
**IL-17A (CTLA-8)**	Interleukin-17A (IL-17A, CTLA-8) is a CD4+ T cell-derived cytokine that promotes inflammatory responses
**IP-10 (CXCL10)**	IP-10 has no activity on neutrophils. Its functions include stimulation of monocytes, natural killer and T-cell migration, regulation of T-cell and bone marrow progenitor maturation, modulation of adhesion molecule expression as well as inhibition of angiogenesis.
**MCP-1 (CCL2)**	It is involved in the recruitment or migration of leukocytes, more specifically monocytes, to the sites where the inflammatory response is taking place.
**MIP-1 alpha (CCL3)**	Macrophage inflammatory protein-1 alpha (MIP-1α/CCL3) is a chemotactic chemokine secreted by macrophages. MIP-1α/CCL3 recruits macrophages, lymphocytes, and eosinophils via the CCR1 or CCR5 receptor. MIP-1α/CCL3 preferentially attracts activated CD8+ T cells.
**MIP-1 beta (CCL4)**	CCL4 recruits and stimulates various inflammatory cells at sites of inflammation. CCL4 is produced by lymphocytes, macrophages, and dendritic cells. Both CCL4 and the related protein CCL3 participate in the host response to invading bacterial, viral, parasite and fungal pathogens by regulating the trafficking and activation state of selected subgroups of inflammatory cell.
**TNF alpha**	TNF alpha is a multifunctional proinflammatory cytokine that belongs to the tumor necrosis factor (TNF) superfamily. This cytokine is mainly secreted by macrophage and bind to its receptors, TNFRSF1A/TNFR1 and TNFRSF1B/TNFBR. TNF alpha is involved in the regulation of immune cells, cell proliferation, differentiation, apoptosis, lipid metabolism, and coagulation.

SUPPLEMENTARY RESULTS:

Supplementary Figure S1: Kaplan-Meier Curves Demonstrating Survival Across FLARE Groups in (A) Breast Cancer; (B) Genitourinary Cancer; (C) Gastrointestinal Cancer; (4) Thoracic Cancer.

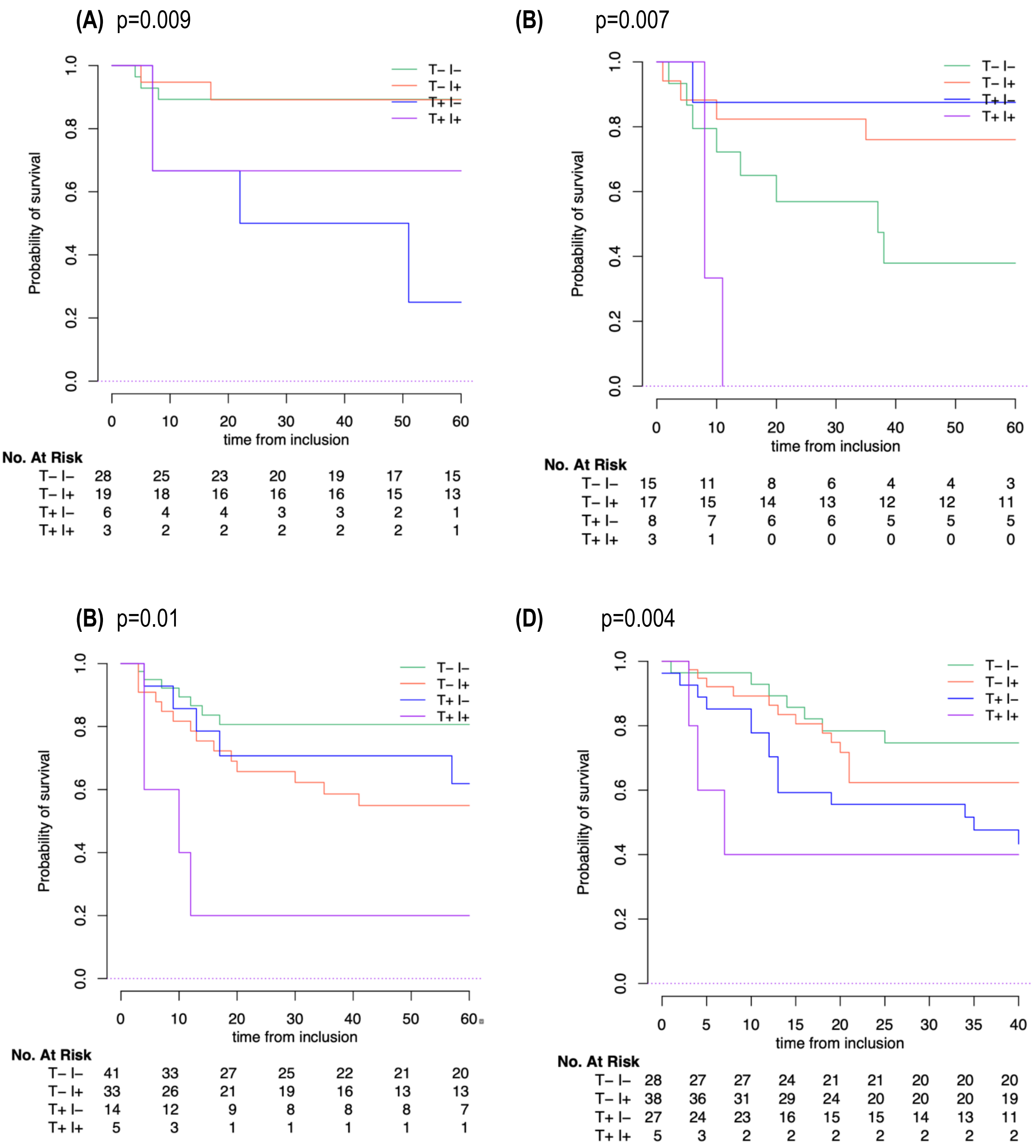

Supplementary Table S4: Baseline characteristics of the study population.

	**Retrospective cohort (n=524)**	**Prospective cohort (n=27)**
**Age** (median; range)	69 (35-98)	65 (49-82)
**Gender**		
Male	271 (52%)	13 (48%)
Female	253 (48%)	14 (52%)
**Smoking**		
Current or former smoker	271 (55%)	19 (70%)
**ECOG Performance Status ≤1**	377 (78%)	15 (56%)
**Comorbidity**		
Hypertension	254 (49%)	14 (52%)
Cardiovascular disease	106 (20%)	4 (15%)
**Cancer-type**		
Thoracic	134 (26%)	5 (19%)
Genitourinary	71 (14%)	7 (26%)
Gastrointestinal	124 (24%)	6 (22%)
Breast	101 (19%)	4 (15%)
**Advanced stage**	334 (64%)	22 (82%)
**Systemic therapy**	299 (57%)	24 (89%)
Chemotherapy	184 (62%)	17 (63%)
Immunotherapy	42 (14%)	4 (15%)
**Covid-symptoms at diagnosis**		
Fever	352 (68%)	21 (78%)
Cough	274 (53%)	10 (37%)
Dyspnea	247 (48%)	9 (33%)
**Covid-treatment**		
Antibiotics	374 (74%)	18 (67%)
Antiviral therapy	112 (31%)	15 (56%)
Steroids	117 (23%)	15 (56%)
Immunomodulators	39 (10%)	9 (33%)
**Median hospital stay duration** (range)	13.5 (1-73)	17 (3-71)
**Admission to ICU**	41 (12%)	4 (15%)
**Severe acute respiratory failure**	115 (25%)	5 (19%)
**Covid-19 complications**	310 (68%)	8 (30%)
**30-day mortality**	141 (29%)	4 (15%)

Supplementary Figure S2: Immunophenotypic Characterization of Immature and Mature Neutrophil Subpopulations

Surface marker	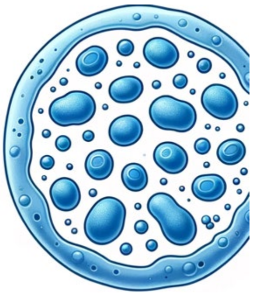 Mature neutrophil	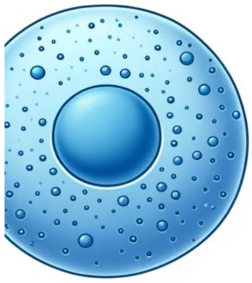 Immature neutrophil
**CD10**	+	-
**CD16**	+	+
**CD11b**	+/-	+/-
**CD15**	+/-	+/-

Supplementary Figure S3: Principal Component Analysis (PCA) of Immature (A) and Mature (B) Circulating Neutrophils Stratified by COVID-19 Severity

(A)

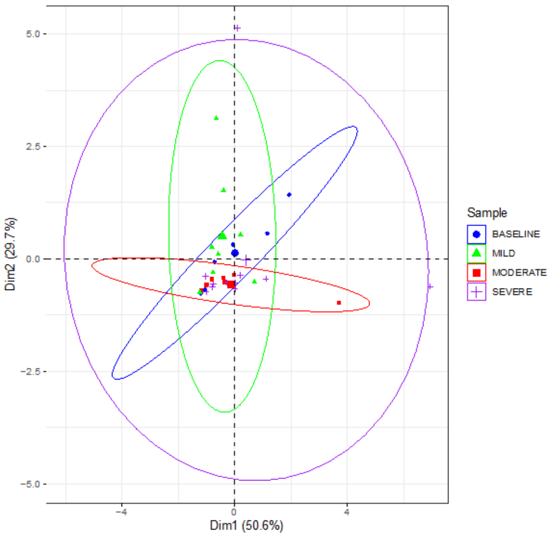

(B)

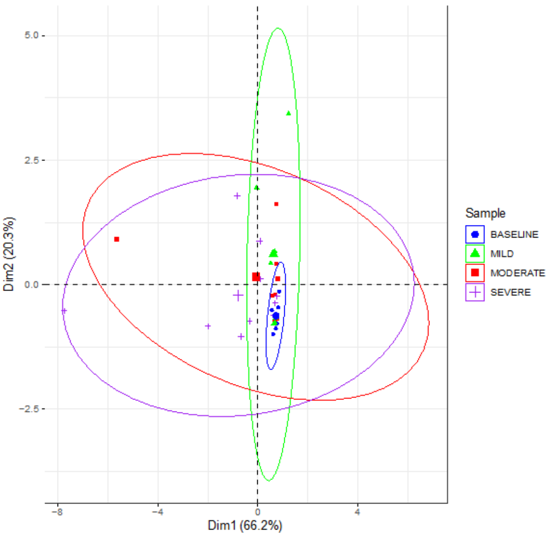

Supplement: Supplementary file 1 [file cancers-16-02974-s001.zip › cancers-3128616-supplementary.pdf]
